# Current and future ozone risks to global terrestrial biodiversity and ecosystem processes

**DOI:** 10.1002/ece3.2568

**Published:** 2016-11-21

**Authors:** Jürg Fuhrer, Maria Val Martin, Gina Mills, Colette L. Heald, Harry Harmens, Felicity Hayes, Katrina Sharps, Jürgen Bender, Mike R. Ashmore

**Affiliations:** ^1^ AgroscopeClimate/Air Pollution GroupZurichSwitzerland; ^2^ Department of Chemical and Biological EngineeringUniversity of SheffieldSheffieldUK; ^3^ Centre for Ecology and HydrologyEnvironment Centre WalesBangorGwyneddUK; ^4^ Department of Civil and Environmental Engineering and Department of Earth, Atmospheric and Planetary SciencesMassachusetts Institute of TechnologyCambridgeMAUSA; ^5^ Institute of BiodiversityThünen InstituteBraunschweigGermany; ^6^ Stockholm Environment InstituteUniversity of YorkYorkUK

**Keywords:** air pollution, atmospheric feedback, Community Earth System Model, G200 ecoregions, global climate change, species diversity

## Abstract

Risks associated with exposure of individual plant species to ozone (O_3_) are well documented, but implications for terrestrial biodiversity and ecosystem processes have received insufficient attention. This is an important gap because feedbacks to the atmosphere may change as future O_3_ levels increase or decrease, depending on air quality and climate policies. Global simulation of O_3_ using the Community Earth System Model (CESM) revealed that in 2000, about 40% of the Global 200 terrestrial ecoregions (ER) were exposed to O_3_ above thresholds for ecological risks, with highest exposures in North America and Southern Europe, where there is field evidence of adverse effects of O_3_, and in central Asia. Experimental studies show that O_3_ can adversely affect the growth and flowering of plants and alter species composition and richness, although some communities can be resilient. Additional effects include changes in water flux regulation, pollination efficiency, and plant pathogen development. Recent research is unraveling a range of effects belowground, including changes in soil invertebrates, plant litter quantity and quality, decomposition, and nutrient cycling and carbon pools. Changes are likely slow and may take decades to become detectable. CESM simulations for 2050 show that O_3_ exposure under emission scenario RCP8.5 increases in all major biomes and that policies represented in scenario RCP4.5 do not lead to a general reduction in O_3_ risks; rather, 50% of ERs still show an increase in exposure. Although a conceptual model is lacking to extrapolate documented effects to ERs with limited or no local information, and there is uncertainty about interactions with nitrogen input and climate change, the analysis suggests that in many ERs, O_3_ risks will persist for biodiversity at different trophic levels, and for a range of ecosystem processes and feedbacks, which deserves more attention when assessing ecological implications of future atmospheric pollution and climate change.

## 1 Introduction

Declining biodiversity is a global concern, and pressures from human influences such as land use and changing environmental conditions, including atmospheric nutrient inputs and climate change, are expected to persist in the future (Sala et al., 2000). This has wide implications for ecosystem function (Hooper et al., 2012) and, in turn, for provisioning multiple ecosystem services to humans (Cardinale et al., 2012). Changing habitat conditions and disturbance are among the main causes of changes in plant communities at a global scale (Tilman & Lehman, 2001). Air pollution is recognized as an important factor affecting habitat conditions globally, while tropospheric ozone (O_3_) has been identified as the most widespread phytotoxic gaseous pollutant causing significant long‐term abiotic stress over large areas (Ashmore, 2005). As a result of increasing emissions of precursor gases (carbon monoxide [CO], oxides of nitrogen [NO_x_], volatile organic compounds [VOC], and methane [CH_4_]), mean concentrations have been growing since the 1950s, at a rate of 5 ppb/decade on average in the northern hemisphere (NH) and by 2 ppb/decade in the southern hemisphere (SH; Cooper et al., 2014). According to the four representative concentration pathways (RCPs) used in the Intergovernmental Panel on Climate Change Fifth Assessment Report (AR5; IPCC, 2013), by the middle of this century, both increases and decreases in tropospheric O_3_ concentrations are possible, depending on the regional balance between processes leading to either formation or destruction of O_3_, and the extent of adoption of air pollution abatement measures underlying the different RCPs (Fiore et al., 2012). Greenhouse gas emissions differ between RCPs, and the consequent effects on climate and land use also alter the concentrations and distribution of O_3_, which also acts as important greenhouse gas.

The global threats to agricultural yields and food security posed by O_3_ under different scenarios have been quantified and discussed by several studies (Chuwah, van Noije, van Vuuren, Stehfest, & Hazeleger, 2015; Tai, Martin, & Heald, 2014). In contrast, implications for biodiversity at the global scale are much less certain and have had little recognition. This is an important gap, which deserves attention when assessing ecological implications of future developments of atmospheric pollution and climate. Here, we provide a global evaluation of the current (year 2000) and future (2050) O_3_ exposure of the Global 200 (G200) terrestrial ecoregions (ER), which are priority regions for conservation (Olson & Dinerstein, 1998; wwf.panda.org). ERs have relatively uniform climate with a characteristic set of ecological communities. They are typified by high numbers of endemic species, high taxonomic uniqueness, global rarity, and/or unique ecological phenomena. They have been selected for their irreplaceability and distinctiveness and represent all the major global biomes. We focus on the G200 ERs, rather than on biodiversity hot spots, because our focus is on broader issues of ecosystem structure and function, rather than the threat to individual species.

We link this evaluation of O_3_ exposure to a critical focused review of the observational and experimental evidence for impacts of elevated O_3_ exposure on terrestrial biodiversity, and on downstream ecosystem processes and related feedbacks to the atmosphere. This review is based mostly on evidence in temperate regions, and we discuss the extrapolation to regions for which little knowledge of O_3_ effects currently exists. Finally, we assess possible risks and benefits of different climate and air pollution policies for the ERs, and for the major biomes within which they are situated, in different regions of the world.

Our simulations used the Community Earth System Model (CESM; Appendix S1), including changes in anthropogenic emissions of precursor gases and climate, but not land use (Val Martin et al., 2015). The CESM model reproduces global surface O_3_ levels well, although values at any location may differ by up to 15% from measured values (Tilmes et al., 2016). We considered results for two contrasting scenarios (Table S1): RCP4.5, which aims to stabilize global radiative forcing at 4.5 W/m^2^ by the end of the century, and RCP8.5, in which greenhouse gas emissions continue to increase over this century, and there is no climate stabilization. The global precursor emissions of CO, NO_x_, and VOCs in 2050 are similar in these two RCPs, but 2050 concentrations of CH_4_ are much higher under RCP8.5; this is relevant because CH_4_ contributes to background tropospheric O_3_ levels both as an O_3_ precursor and by its effect on global warming (West & Fiore, 2005).

## 2 O_3_ Exposure of G200 Ecoregions

**Figure 1:**
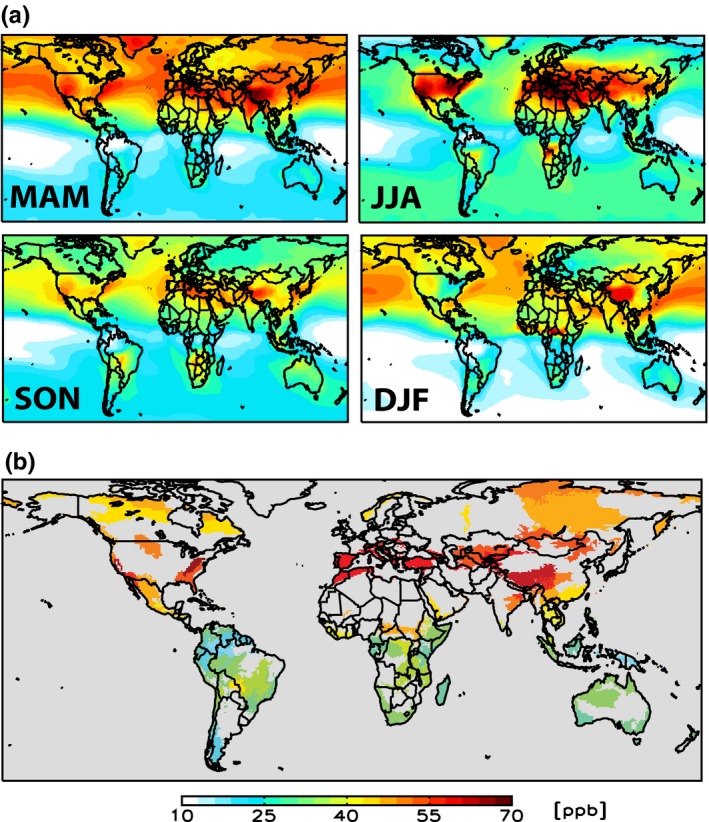
Simulated surface O_3_ concentrations in 2000. (a) Seasonal daily 12‐hour (M12) averages (from 6 a.m. to 6 p.m. LST) for March–April–May (MAM), June–July–August (JJA), September–October–November (SON), and December–January–February (DJF). (b) Simulated maximum M12 (i.e., the highest of the four seasonal values in (a)) within G200 ER. The map shows CESM M12 output (1.9 × 2.5°) regridded to the G200 map resolution (0.25 × 0.25°). M12 concentrations outside the G200 areas are masked in gray

From the CESM simulations of hourly surface O_3_ concentrations, we derived a metric designed to capture the risk of long‐term ecological damage associated with O_3_ exposure. This was the M12 exposure index, representing the mean 12‐hr daylight concentration over a three‐month period. The global distribution of M12 for four‐three‐month periods in 2000 reveals that in the March–August period, highest O_3_ exposures are found at mid‐latitudes in NH (Figure 1a). Concentrations are similarly distributed, but lower in the NH in September–November, while in December–February, concentrations are highest in areas of China and moderate over more southern landmasses. In the SH, where O_3_ concentrations are generally lower, the highest values are found in mid‐latitudes from June to November.

In order to assess the risk for individual G200 ERs, we calculated the mean value of each three‐month M12 value over the whole area of each ER. We then selected the highest of the four three‐month M12 means, avoiding seasons with no active vegetation at high latitudes. We used this index, rather than the highest running mean three‐month M12, so that we could relate individual ER exposure to the global distribution of exposure in the same fixed periods. Figure 1b shows the resultant ER exposure in 2000. This highlights the relatively low exposure of the SH and tropical ERs compared with those in the NH from the subtropics to the poles. It is important to emphasize that this is an indicative measure of risk—within each ER, there will be spatial variation in both O_3_ exposure and O_3_ uptake and plant community distribution which cannot be assessed at this global scale.

The UNECE Convention on Long‐Range Transboundary Air Pollution (CLRTAP) uses AOT40 as an exposure index for estimating ecological risks (Appendix S1) and defines an AOT40 of 3 ppm hr accumulated over 3 months as a critical level of O_3_ “above which adverse effects may occur on the growth of the most sensitive species of (semi‐) natural communities dominated by annuals” (CLRTAP, 2015). We estimate that at a three‐month mean M12 value of 35–40 ppb, there is a high likelihood that the AOT40 critical level will be exceeded (Figure S1).

The three‐monthly M12 values in individual G200 ERs ranged from 15.8 to 65.2 ppb (Table S2). The 10 ERs with the highest exposure all have maximum three‐monthly M12 values ≥60 ppb (Table 1). These ERs fall within five major biomes; four are in the temperate broadleaf and coniferous forests biome, two in the Mediterranean forests, woodlands, and shrubs biome, and three are in the montane grasslands biome; only the Terai‐Duar savannas and grasslands, at the base of the Himalayas, are in a subtropical biome. Three are in North America, five in Asia, and two stretch across Eurasia. Some of these ERs are also associated with global biodiversity hot spots, as identified by Myers, Mittermeier, Mittermeier, de Fonesca, and Kent (2000), for example, in California, the Mediterranean Basin, the Caucasus, and the Himalayas. As CESM model predictions may differ from measured values (see above), there is some uncertainty in the precise rankings of ERs in Table 1, and in Table S2, which provides M12 values for all ERs.

**Table 1:** G200 ERs with the highest simulated monthly M12 exposure (ppb) in 2000

Biome	ER name	M12
Temperate broadleaf and mixed forests	Appalachian and mixed mesophytic forests	65.2
Mediterranean forests, woodlands, and shrub	California chaparral and woodlands	65.0
Temperate broadleaf and mixed forests	Western Himalayan temperate forests	64.3
Montane grassland and shrublands	Tibetan Plateau steppe	63.9
Tropical and subtropical grasslands savannas and shrublands	Terai‐Duar savannas and grasslands	62.4
Temperate coniferous forests	Sierra Nevada coniferous forests	62.0
Montane grassland and shrublands	Eastern Himalayan alpine meadows	61.1
Mediterranean forests, woodlands, and shrubs	Mediterranean forests	60.8
Temperate coniferous forests	Caucasus‐Anatolian‐Hyrcanian temperate forests	60.2
Montane grassland and shrublands	Middle Asian montane steppe and woodlands	59.8

## 3 Field Evidence of O_3_ Injury

Phytotoxic effects of elevated O_3_ exposures depend on molecular diffusion of the gas into plants via the stomatal pores on the leaf surface. Inside leaves, O_3_ reacts with unsaturated biomolecules to form reactive oxygen species initially causing programmed cell death, visible on the leaf surface as small necrotic lesions (Vainonen & Kangasjärvi, 2015). Such visible injury is the only indicator of adverse impacts that can be routinely surveyed in the field. Records gathered between 2007 and 2015 from different sources of 500 observed incidences of visible O_3_ injury under ambient conditions, and verified by experts (Appendix S1), revealed that over 80% of these incidences, involving 67 species of forbs, shrubs, and trees across four continents, occurred in Europe and North America where studies on O_3_ effects on vegetation tend to be concentrated, but visible injury was also reported for Asia and Latin America despite lower monitoring effort there (Table 2). Not all these incidences or species were within G200 ERs, but this information confirms the potential for O_3_ impacts in many world regions. This is supported by other studies; for example, in Europe, 95 species of grasses, forbs, and shrubs exhibited visible O_3_ injury over the period 1990–2006 (Mills, Hayes, et al., 2011; Mills, Pleijel, et al., 2011), and in forests, a systematic assessment of observational data for 2009 revealed symptoms in 17 different species across 13 European countries (Schaub & Calatayud, 2013).

**Table 2:** Number of incidences of recorded ozone injury, by continent, observed between 2007 and 2015

Region	Forb	Shrub	Tree	Forb, shrub and tree	Total
Europe	25	65	54		144
N America	42	1	3	244	290
S America	9	No data	5		14
South‐East Asia	14	10	33		57
Total	90	76	95		505

Total number of species of forb, shrub and tree injured = 67. Data on “Forbs, shrubs and trees” for North America is from a summary report of visible O_3_ injury records from 4333 visits to O_3_ biomonitoring sites across the continent between 2007 and 2010 (U.S. Environmental Protection Agency, 2014). Each site has at least 30 individual plants of two bioindicator species present. The list of species includes a variety of relatively common forbs, shrubs and herbs, which are easy to identify.

Despite this evidence of exposure of many ERs to O_3_ above phytotoxic levels, and the evidence of widespread visible injury, O_3_ is largely ignored in global assessments of threats to biodiversity, for instance in the latest assessment by the UN Convention on Biological Diversity “Global Diversity Outlook 4” (CBD, 2014). Furthermore, while Target #8 of the Aichi Biodiversity Targets under the CBD aims to reduce pollution by 2020 to levels that are not detrimental to ecosystem function and biodiversity, there is to date no specific reference to O_3_ and no indicator has been identified. This situation likely reflects the lack of unequivocal evidence of widespread and major alterations in biodiversity due to O_3_ under natural conditions. We suspect that this is because long‐term effects of O_3_ may be subtle and difficult to detect under complex and variable field conditions, including the presence of overlapping factors influencing biodiversity, and we review these issues below.

## 4 Changes in Plant Communities

Some species are better protected from O_3_ stress than others due to differences in leaf diffusive properties, cellular detoxification capacity, compensatory biomass production and allocation, or isoprene emission. However, the genetic basis for the differential sensitivity remains elusive, although some recent studies with O_3_‐sensitive and O_3_‐resistant *Arabadopsis thaliana* (Xu et al., 2015) and crops such as rice (Frei, 2015) have identified multiple qualitative trait loci potentially involved in regulating the O_3_ response. Furthermore, and in contrast to many other environmental stresses, there is a limited functional pattern to O_3_ sensitivity. For example, a meta‐analysis of collated data from field chamber experiments, mainly conducted on grasslands, heathlands, and wetlands in temperate regions of Europe, revealed that, although species with a therophytic life form appear to be generally more sensitive to O_3_, there was no relationship between O_3_ sensitivity and leaf longevity, flowering season, stomatal density or maximum altitude, nor between O_3_ sensitivity and Grime's functional types (Hayes, Jones, Mills, & Ashmore, 2007). However, another analysis (Jones, Hayes, Mills, Sparks, & Fuhrer, 2007) of the same database suggested that light‐loving species tend to be more sensitive than those that normally occur in the shade, plants of dry sites tend to be more sensitive than those found in more moist soils, and plants tolerant of moderately saline conditions are more sensitive than those of nonsaline habitats. The extent to which these findings can be generalized to species in other ERs is uncertain, as the sensitivity of the species in many of the ERs with high O_3_ exposure is unknown. But the fact that species from the Fabacea (or Leguminosae) family have consistently been found to be relatively more sensitive than those of other families, and because Fabacea, including many trees, shrubs, and herbaceous plant species, are an ubiquitous component of both temperate and tropical ERs, O_3_‐sensitive species are likely to be present in ERs that so far have not been monitored.

The existence of wide differences in sensitivity between species implies that O_3_ stress can cause long‐term shifts in species evenness or richness in diverse plant communities. There is some observational evidence of such effects within the North America ERs listed in Table 1; for instance, changes in species richness in coastal shrub vegetation (*Artemisia californica* Less.) within the “California chaparral and woodlands” ER were attributed to O_3_ (Westman, 1979), and significant changes in stand composition have been reported along O_3_ gradients in the San Bernardino Mountains (Miller, 1973), within the “Sierra Nevada coniferous forest” ER (Arbaugh & Bytnerowicz, 2003), although effects of O_3_ in these areas may be difficult to separate from other influencing factors such as high nitrogen (N) deposition (Fenn, Poth, Bytnerowicz, Sickman, & Takemoto, 2003). Payne et al. (2011) identified O_3_ as a key driver of compositional changes in species in British acid grassland, in addition to N deposition, although it was not associated with a reduction in species richness or diversity indices. In general, field evidence for compositional changes remains very scarce, and most evidence for the potential impact of O_3_ on plant diversity therefore rests on data from controlled experiments with either artificial model communities or intact ecosystems in which O_3_ levels are varied while other factors are kept constant (Weigel, Bergmann, & Bender, 2015).

Compositional changes caused by O_3_ remain difficult to predict from the observed responses of individual species when grown alone (Bassin, Volk, & Fuhrer, 2007). Likely reasons for that are species interactions in terms of competition or facilitation. Competition dominates under low levels of stress, while facilitation becomes more important under increasing stress and decreasing productivity (Maestre, Callaway, Valladares, & Lortie, 2009). Consequently, species responses inside communities can be contrary to expectations. For example, in a grassland community, O_3_‐sensitive forbs benefited from elevated O_3_, due to reductions in the cover of dominant grass species (Evans & Ashmore, 1992). Exposure to elevated O_3_ of an upland mesotrophic grassland in the UK that was managed to increase species diversity significantly decreased the biomass of *Ranunculus* species; this was attributed to reduced performance of the hemi‐parasitic species *Rhinanthus minor* (yellow rattle), a species that reduces the productivity of grasses and opens up the grassland canopy, suggesting that O_3_ stress may be a significant barrier to achieving increased species diversity in managed grasslands because of its effects on this keystone species (Wedlich et al., 2012). In an early succession pine forest community, O_3_‐sensitive blackberry (*Rubus cuneifolius*) reached the highest cover under high O_3_ exposure (Barbo, Chappelka, Somers, Miller‐Goodman, & Stolte, 1998), either because growth of blackberry was less affected by O_3_ than its leaf injury indicated, or it was more effective in out‐competing other, less O_3_‐sensitive species for resources. In general, effects of O_3_ on the competitive balance between species are not uniform and may depend on the species mixture (Nussbaum, Bungener, Geissmann, & Fuhrer, 2000).

In herbaceous species, short‐term sensitivity of growth to O_3_ is positively related to inherent relative growth rate (Bungener, Nussbaum, Grub, & Fuhrer, 1999; Danielsson, Gelang, & Pleijel, 1999; Davison & Barnes, 1998) suggesting that faster growing species tend to be more O_3_‐sensitive than slower growing species. Thus, in ERs where relative growth rates are generally low, O_3_ stress would be less damaging than in ERs dominated by faster growing species. In fact, after several years, changes in the functional group composition of subalpine grassland at high O_3_ (Volk, Bungener, Contat, Montani, & Fuhrer, 2006) could not be separated statistically from nutrient gradient effects (Stampfli & Fuhrer, 2010). Similarly, a montane Geo‐Montani‐Nardetum proved resilient to long‐term O_3_ exposure, regardless of extra N input (Bassin, Volk, & Fuhrer, 2013); this was not caused by low canopy O_3_ uptake (Volk, Wolff, Bassin, Ammann, & Fuhrer, 2014). However, in the absence of changes in species, micro‐evolutionary adaptation to O_3_ stress might be involved in these permanent old grasslands (Kölliker, Bassin, Schneider, Widmer, & Fuhrer, 2008) and also in some forests (Moran & Kubiske, 2013). For instance, because of a competitive disadvantage, the most sensitive aspen genotype was eliminated in a seven‐year exposure to elevated O_3_ from the seedling stage through to maturity, although total growth of the stand was not affected (Kubiske, Quinn, Marquardt, & Karnosky, 2007).

Shifts in community composition could also result from specific changes in reproductive success caused by decreased biomass allocation (Bender, Bergmann, & Weigel, 2006; Wang et al., 2015) impairing reproductive growth and development (Leisner & Ainsworth, 2012) and seed production (Bender et al., 2006; Harward & Treshow, 1975), or from direct effects of O_3_ on reproductive structures (Black, Black, Roberts, & Stewart, 2000). In temperate grasslands, experimental O_3_ treatment reduced seed number, fruit number, and weight, but increased flower number and flower weight in a number of species, for example, in paper birch (*Betula papyrifera*) (Leisner & Ainsworth, 2012), and decreased seed weight and germination rate (Darbah et al., 2008) with implications for the establishment and survival of the progeny. Where plant composition greatly depends on the belowground seed pool, declining reproductive success can be caused by elevated O_3_ exposure, such as in the Dehesa annual grasslands, which cover several million hectares in the Iberian Peninsula within the highly O_3_‐exposed “Mediterranean forests, woodlands, and shrubs” ER (Table 1; Gimeno, Bermejo, Sanz, De La Torre, & Elvira, 2004).

Effects of O_3_ stress at the community level can be masked by interaction with disturbances caused by pests and diseases. Altered leaf surface properties increased the natural infection by leaf rust (*Melampsora medusae* Thuem. f. sp. *tremuloidae*) in trembling aspen (*Populus tremuloides* Michx.) (Percy, Mankovska, Hopkin, Callan, & Karnosky, 2003), and induced changes in host plant preferences, thus altering the distribution of herbivory, as well as competitive interactions among them (Agrell, Kopper, McDonald, & Lindroth, 2005). O_3_ stress improved tree foliage quality for herbivores and thus favored the growth of leaf‐chewing insects (Valkama, Koricheva, & Oksanen, 2007) as many specialist insect herbivores succeed well on diets containing material with a high level of phenolics or terpenoids. Also, O_3_ can affect pollination and food supply of nectar‐feeding insects through changes in flowering timing and signaling. Flowering can be delayed, as in *Campanula rotundifolia* and *Vicia cracca* in a northern meadow community (Rämö, Kanerva, Ojanperä, & Manninen, 2007), or accelerated, as in *Lotus corniculatus* in calcareous grassland (Hayes, Wagg, Mills, Wilkinson, & Davies, 2012; Hayes, Williamson, & Mills, 2012), and such subtle shifts play an important role when flowering is closely synchronized with pollinating species (Black et al., 2000). In addition, floral scent trails in the form of VOCs emitted by flowers that are essential for plant‐insect interactions are chemically degraded or transformed by O_3_ (Blande, Holopainen, & Niinemets, 2014; Farré‐Armengol et al., 2016), thus reducing the signaling distance and the signal specificity and efficiency (McFrederick, Fuentes, Roulston, Kathilankal, & Lerdau, 2009); in turn, in patchy or fragmented habitats, pollinators spend more time searching for flowers (McFrederick, Kathilankal, & Fuentes, 2008).

## 5 Changes in Soil Microbiota and Nutrient Cycling

**Figure 2:**
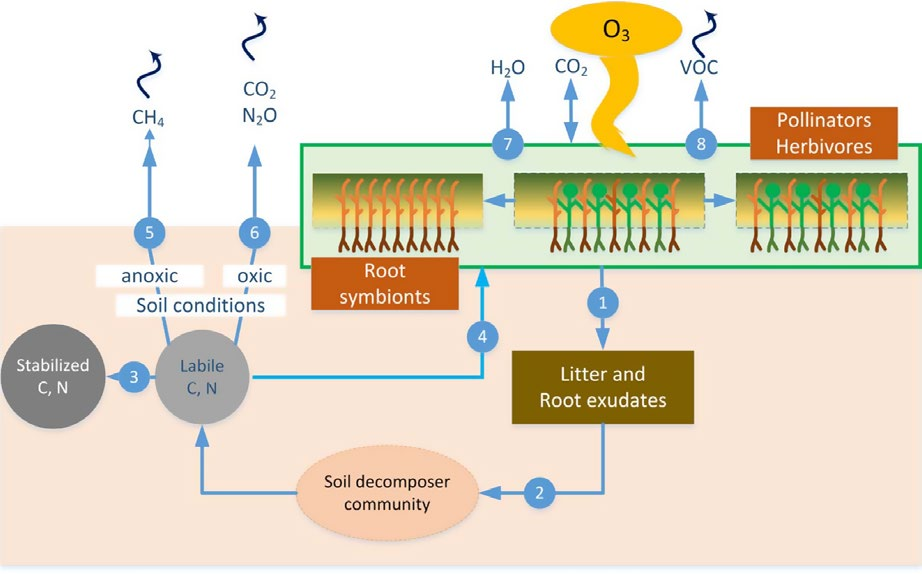
Diagram summarizing main downstream processes affected by O_3_ uptake in plant communities, starting either with or without changes in species composition (box), and ultimately feeding back to atmospheric composition. 1, Reduced litter input and root exudation, lower degradability; 2, altered microbiota and slower decomposition; 3, increased immobilization of C and N; 4, reduced nutrient availability; 5, altered methanogenic activity in wetlands; 6, reduced soil respiration and N availability for denitrification; 7, loss of water flux control under drought; 8, emission of biogenic volatile organic compounds

The belowground ecosystem compartment is insulated from direct O_3_ exposure, but there is an accumulating body of evidence that effects aboveground translate into changes in soil microbial communities, and further propagate through the microbial food web to alter carbon (C) and N cycling (Lindroth, 2010). The main pathways considered here, and their implications for ecosystem processes, and feedbacks to the atmosphere, are depicted in Figure 2.

A general, but highly variable, trend is that under high O_3_, relatively less biomass is allocated to roots compared to shoots, with a mean reduction by 5.6% across all species covered in a meta‐analysis (Grantz, Gunn, & Vu, 2006). This reduces the amount of root detrital inputs and consequently may significantly affect long‐term soil C formation rates (Loya, Pregitzer, Karberg, King, & Giardina, 2003). In addition to litter input, litter decomposition is a key process in nutrient cycling, which in complex ways depends on the diversity of litter, the decomposer community (Gessner et al., 2010), and environmental and soil conditions. It has been suggested that plant species richness is not related to the diversity of litter composition (Meier & Bowman, 2008), and thus, O_3_ effects at the species diversity level may be of limited relevance for litter decomposition in the soil. But, evidence exists that, in the absence of changes in plant diversity, O_3_ slows decomposition, although a general pattern is lacking and a range of different mechanisms could be involved (Couture & Lindroth, 2013).

Slower decomposition could be related to changing soil microbial functional diversity caused by altered litter quality (Aneja et al., 2007). Litter from O_3_‐exposed plants is more recalcitrant (Kim, Chappelka, & Miller‐Goodman, 1998) due to a higher C/N ratio (Wittig, Ainsworth, Naidu, Karnosky, & Long, 2009), a higher level of tannins and related phenolic compounds (Liu, King, & Giardina, 2005), and more lignin (Richet et al., 2012). Soil fauna also plays an essential role in recycling of soil organic matter (SOM), energy, and nutrients. There is evidence of negative effects of O_3_ on soil nematodes (Bao, Li, Hua, Zhao, & Liang, 2014), collembolans, enchytraeids, and soil mites (Schrader, Bender, & Weigel, 2009), which could further slow decomposition. In the long run, reduced degradability of litter leads to increased immobilization of C and N in recalcitrant soil fractions, as observed in soils of forests (Holmes, Zak, Pregitzer, & King, 2006) and montane grassland (Bassin et al., 2015), which feeds back to plants via altered nutrient availability. However, such effects are subtle and vary across sources of litter and environmental conditions.

Decomposition is often positively related to residue N content (Hobbie et al., 2012), but contrasting results have been reported for the impact of O_3_ on litter N concentration. Whereas King, Liu, and Aspinwall (2013) reported that elevated O_3_ causes a general decrease in litter N concentration, others have found an increase (Lindroth et al., 2001), which may explain why litter decomposition differs between species (Williamson, Mills, & Freeman, 2010). Decomposition of SOM by fungi decreases in elevated O_3_ (Edwards & Zak, 2011; Yue et al., 2015) more than decomposition by bacteria (Zhang et al., 2014), suggesting that any effect of O_3_ on decomposition could be lower in productive systems with soil communities dominated by highly active bacteria than in systems with lower productivity where fungi and less active bacteria dominate.

Reduced C allocation to roots impairs mycorrhizal symbiosis, for instance, in birch (Kasurinen et al., 2005), hybrid aspen (Edwards & Zak, 2011), beech (Pritsch et al., 2009), hybrid larch (Wang et al., 2015), and blue wild rye (*Elymus glaucus*; Yoshida, Gamon, & Andersen, 2001). Mycorrhizae are ubiquitous in all terrestrial ecosystems and play an essential role in soil–plant nutrient exchange and via the turnover of external mycelium for the transfer of root‐derived C to SOM (Godbold et al., 2006). Lower O_3_ stress would thus not only benefit ectomycorrhizal diversity and richness (Katanić, Paoletti, Orlović, Grebenc, & Kraigher, 2014), but also soil nutrient and C cycling, particularly in very dry, wet, or cold habitats where plant productivity is limited by environmental conditions, such as those at high latitudes or in montane regions.

## 6 Implications for Terrestrial Feedbacks to the Atmosphere

Lindroth (2010) suggested that O_3_ affects belowground communities and ecosystems processes primarily via reduced quantities of litter produced, with implications for the net exchange of CO_2_ between ecosystems and the atmosphere, in agreement with Chapman, King, Pregitzer, and Zak (2005) who concluded that changes in soil C cycling are most likely be brought about by changes in litter production rather than quality. The combination of reduced plant productivity and lower relative biomass allocation to roots (Andersen, 2003) limits the transfer of labile C litter input to soils (Kanerva, Palojärvi, Rämö, & Manninen, 2008), as discussed above. Hence, less O_3_ stress could positively affect C inputs to soils and thus contribute to the protection or even to the increase in the terrestrial sink for CO_2_ and, consequently, to slowing global warming (Ren et al., 2007; Sitch, Cox, Collins, & Huntingford, 2007). Changed litter quantity was associated with increased microbial respiration (Hillstrom, Meehan, Kelly, & Lindroth, 2010; Kasurinen, Kokko‐Gonzales, Riikonen, Vapaavuori, & Holopainen, 2004; Nikolova, Andersen, Blaschke, Matyssek, & Häberle, 2010), but such an increase might be affected by other environmental factors such as soil water availability (Nikolova et al., 2010). Microbial biomass and soil respiration were not significantly affected by O_3_ in the aspen open‐air exposure study (Larson, Zak, & Sinsabaugh, 2002). As effects of O_3_ on plant chemistry and ecological interactions are highly context‐ and species‐specific, it remains difficult to identify general, global patterns. But from the limited evidence, it can be hypothesized that in spite of lower soil C inputs associated with reduced net primary production, soil C stocks could increase due to lower degradability of the litter and reduced microbial activity.

However, data from sufficiently long O_3_ exposure studies are extremely rare, and findings are variable. In experimental forests, O_3_ reduced the C content in woody tissues and in the near‐surface mineral soil (Talhelm et al., 2014), and in more stable SOM pools (Hofmockel, Zak, Moran, & Jastrow, 2011), but data from a high‐elevation grassland experiment indicated that soil C remains unchanged, possibly because a low C input was compensated by reduced turnover (Volk et al., 2011), as discussed above. Similarly, in a modeling study, the replacement of sensitive by more tolerant plant species or genotypes (as also discussed above) in a temperate deciduous forest led to unchanged biomass C stocks in the long term (>100 years) (Wang, Shugart, Shuman, & Lerdau, 2016). Hence, it remains difficult to identify general, global patterns of effects of changing O_3_ exposure on ecosystem C storage.

There is evidence that O_3_ stress affects CH_4_ emissions from wetlands, similar to rice paddies, for which combined data from three studies suggested a reduction by 40% in CH_4_ emission at an O_3_ level (M12) equivalent to around 48 ppb (Tang, Liu, Zhu, & Kobayashi, 2015). As O_3_ does not directly reach soil methanogenic or methanotrophic organisms, it is likely that altered C allocation to roots and reduced root exudation modifies CH_4_ release via changes in the activity and functional diversity of soil microbial communities (Jones, Freeman, Lloyd, & Mills, 2009). However, there is evidence for an inhibitory effect of O_3_ on CH_4_ emission in temperate and boreal peatlands, but the underlying mechanisms remain unclear. Mörsky et al. (2008) found slightly reduced CH_4_ emissions associated with increased microbial biomass resulting from higher substrate availability. Conversely, Toet, Ineson, Peacock, and Ashmore (2011) observed a significant negative effect of O_3_ in the absence of aboveground effects on dominant species such as *Eriophorum* and *Sphagnum*, and in dissolved organic C, suggesting that belowground changes in rhizodeposition, root turnover, and, importantly, microbial community structure could be responsible for reduced CH_4_ production. Williamson, Mills, Hayes, Jones, and Freeman (2016) found that the effect of O_3_ on CH_4_ emission varies with exposure: moderate short‐term O_3_ exposures increases CH_4_ emissions, whereas higher exposures have negative or no significant effect. Although the available data for temperate and boreal peatlands are limited, and the underlying mechanisms require further study, changes in CH_4_ emissions under increasing O_3_ in northern peatlands could provide important positive or negative feedbacks because of the involvement of CH_4_ in background O_3_ production and global warming (West & Fiore, 2005; Wild et al., 2014).

Impacts of O_3_ on N_2_O emissions are even less certain, but N immobilization due to decreased decomposition not only limits the availability of N for plants, as reviewed above, but also for the denitrifier community, which could reduce the potential for nitrification and denitrification (He et al., 2014; Kou, Cheng, Zhu, & Xie, 2015). Although this may be less relevant in systems with a low soil N status but high species diversity, other factors might contribute to the direction of O_3_ effects on N_2_O emissions, including species richness. Niklaus et al. (2016) reported for grassland that N_2_O emission decreased with increasing species richness, but increased with fertilization and the fraction of legumes in the community. A decline in O_3_‐sensitive legumes would thus be associated with reduced N_2_O emission.

Overall, from the limited data, we conclude that the exchange of important greenhouse gases is sensitive to ecosystem O_3_ exposure, with mostly inhibitory effects of higher O_3_ concentrations, but that the issue warrants further investigations.

## 7 Future Levels of O_3_ Exposure

**Figure 3:**
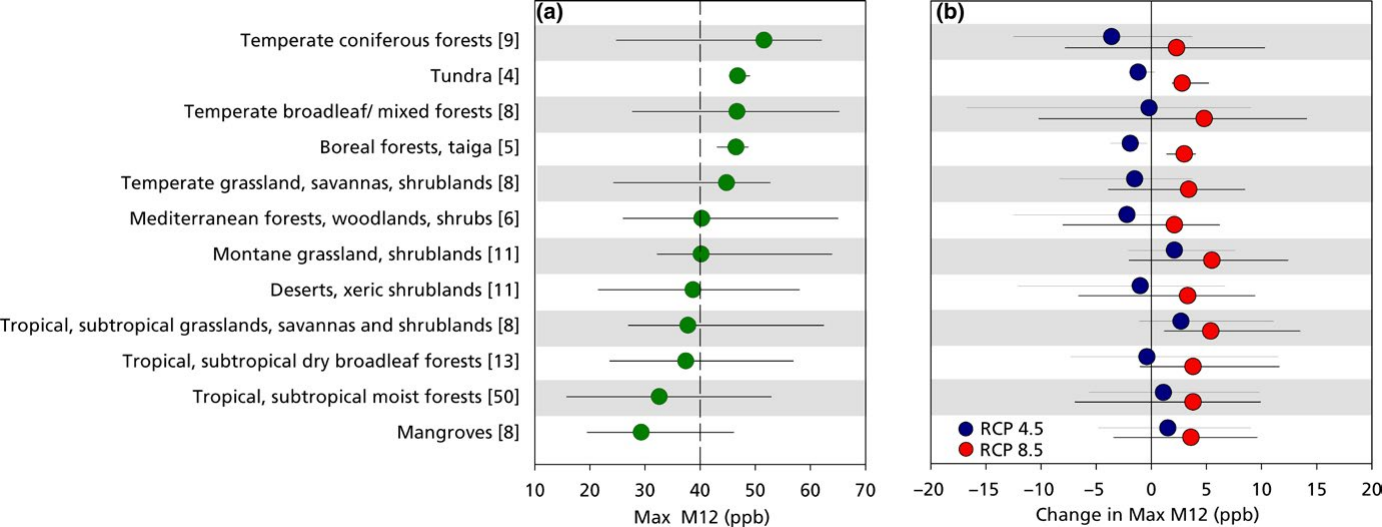
(a) Simulated O_3_ exposure in 2000 in G200 terrestrial ecoregions (ERs), grouped by biome. (b) Change in simulated O_3_ exposure between 2000 and 2050 under RCP4.5 and RCP8.5. ERs are grouped by major biome, and the number of ERs in each biome is shown within brackets. Exposure in (a) is based on the highest of the four seasonal M12 values (Max M12, ppb) in each ER. The dashed line in (a) represents the M12 corresponding to the threshold used to calculate concentration‐based critical levels according to the UNECE CLRTAP. Values are shown for the mean value within the biome (circles) and the minimum/maximum range of values in individual ERs within that biome. Note that the average for the major biomes smoothed out some of the large exposure values shown in Figure 1 for the individual G200 biomes

Many of the effects of O_3_ reviewed here are likely to be slow and may take decades to become detectable, depending on the future trajectory of O_3_ exposure in different ERs. In order to more clearly interpret current and future O_3_ exposure in relation to evidence of effects, we grouped the ERs into 12 major biomes. The range of mean M12 values within each major biome for 2000 is shown in Figure 3a, based again on the maximum of the four seasonal values. About 40% of the ERs had a mean M12 value above 40 ppb, and the critical level set by the UNECE was exceeded in at least one ER within each major biome. The highest mean M12 values, all above 40 ppb, were found in temperate forests and grasslands, boreal forests, and tundra; in contrast, mean M12 values in all the tropical and subtropical biomes were below 40 ppb. The range in M12 values within tundra and boreal forests is small, with no individual ER having a M12 above 50 ppb; these biomes have the smallest number of ERs, and all are in the NH, in regions with a relatively high springtime M12 (Figure 1). All other biomes show a wide range (typically of 25–35 ppb) in individual ERs, largely reflecting the contrasts between NH and SH shown in Figure 1; for example, M12 values for the six ERs in Mediterranean forests, woodlands, and shrubs are in the range 26–32 ppb in South Africa, Chile, and Australia, but are 61 ppb in Europe and 65 ppb in California.

Figure 3b shows the simulated changes in M12 in major biomes, as the range of 2050–2000 differences. The data suggest that under RCP4.5, biome mean M12 declines in 2050 in the temperate and boreal biomes, which had the highest M12 values in 2000, while mean M12 values for tropical, subtropical, and montane biomes tend to increase. Under RCP4.5, the changes in biome mean M12 are relatively small, ranging from −3.6 to +2.7 ppb. Overall, under RCP4.5, exactly 50% of the 142 ERs show an increase in M12, and 50%, a decrease. In contrast, under RCP8.5, all biomes show an increase in mean M12, with values ranging from +2.0 to +5.4 ppb, and in three of the twelve biomes, M12 increases in every ER.

With the exception of tundra and boreal forests, there is a wide variation in the change in M12 among ERs within biomes under both RCP4.5 and RCP8.5, with individual ERs showing both increases and decreases in M12, with a range of over 10 ppb. The individual ERs, which show an increase of over 10 ppb in M12 under RCP8.5, are listed in Table 3(a), while Table 3(b) lists those showing a decrease of over 10 ppb in M12 under RCP4.5. The seven ERs with modeled increases in M12 above 10 ppb under RCP 8.5 all show increased O_3_ exposure even under RCP4.5. All are in forests and grasslands, and in the region covering part of India, the Himalayas, and western China. In contrast, the five ERs with a decrease of over 10 ppb under RCP4.5, which also show a decreased M12 under RCP8.5, are all within North America, covering temperate forests, Mediterranean forests, and desert biomes.

**Table 3:** G200 ecoregions showing either (a) an increase of over 10 ppb in simulated M12 under RCP8.5 or (b) a decrease of over 10 ppb in simulated M12 under RCP4.5

	ER	RCP4.5	RCP8.5
**(a) Biomes with increasing O_3_**			
Temperate broadleaf and mixed forests	Western Himalayan temperate forests	9.0	14.0
Tropical and subtropical grasslands, savannas and shrublands	Terai‐Duar savannas and grasslands	11.1	13.4
Montane grassland and shrublands	Eastern Himalayan alpine meadows	7.6	12.3
Montane grassland shrublands	Tibetan Plateau steppe	5.1	12.1
Temperate broadleaf and mixed forests	Eastern Himalayan broadleaf and coniferous forests	8.2	11.8
Tropical and subtropical dry broadleaf forests	Chota‐Nagpur dry forests	11.5	11.5
Temperate coniferous forests	Hengduan Shan coniferous forests	3.7	10.2
**(b) Biomes with decreasing O_3_**			
Temperate broadleaf and mixed forests	Appalachian and mixed mesophyte forests	−16.7	−10.3
Mediterranean forests, woodlands, and shrubs	California chaparral and woodlands	−12.5	−7.9
Temperate coniferous forests	Southeastern coniferous and broadleaf forests	−12.5	−8.1
Temperate coniferous forests	Sierra Nevada coniferous forests	−12.4	−6.0
Deserts and xeric shrublands	Sonoran‐Baja Deserts	−12.1	−6.7

Values are the difference between 2000 and 2050 in M12 values.

**Figure 4:**
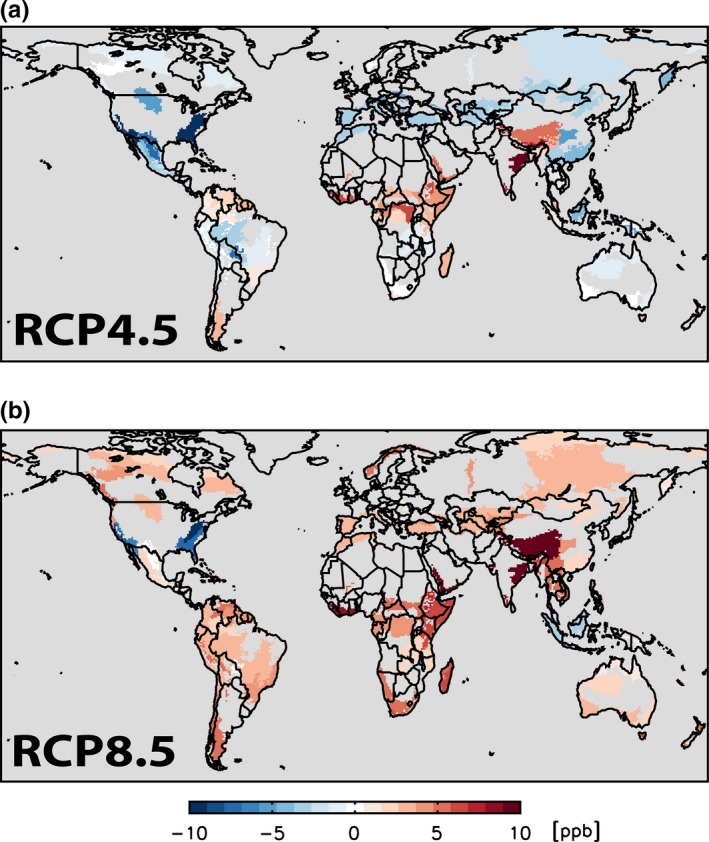
Simulated changes in O_3_ concentration between 2000 and 2050 as a result of the combination of climate and emission changes for RCP4.5 (a) and RCP8.5 (b). Maps show interpolated contours from the 1.9 × 2.5° horizontal resolution output in terms of the change in maximum M12 in G200 ERs. M12 changes outside the G200 areas are masked in gray

The geographical distribution of the changes in M12 exposure of ERs under RCP4.5 and RCP8.5 is shown in Figure 4. The four individual seasonal changes in M12 are shown in Figure S2 (and associated commentary). Under RCP8.5, M12 increases between 2000 and 2050 in almost all ERs, with the greatest increases in South and East Asia; only ERs in North America and parts of South‐East Asia show a decrease in ER exposure. In contrast, under RCP4.5, M12 exposures decrease throughout most of the NH, although increases in M12 are still predicted in the Himalayas, South Asia, sub‐Saharan Africa, and parts of Latin America.

Our model simulations allow us to partition the changes in M12 between the direct effects of changes in anthropogenic precursor emissions and those of climate change alone (Table S3). Increases in M12 values due to climate change alone occur in all biomes except tundra and taiga in the range 0.6–2.1 ppb under RCP4.5 and 1.0–2.9 ppb under RCP8.5, a relatively small difference. However, there are individual ERs in these biomes under both scenarios with a decreased M12 value attributable to climate change; these are mainly island ERs or those close to the coast. The effects of changes in precursor emissions are more variable, both between biomes, and between scenarios. Under RCP4.5, eight of the 12 biomes have reduced mean values of M12, with a maximum reduction of 5.2 ppb in temperate forests; the four biomes with an increase in mean M12 are mainly at low latitudes, with a maximum increase of 1.3 ppb in tropical and subtropical grasslands. In contrast, under RCP8.5, 10 of the 12 biomes have an increased mean M12, with values ranging from 1.9 to 4.1 ppb; only temperate coniferous forests and Mediterranean forests show a very small decrease due to changes in emissions.

## 8 Interactions with Other Abiotic Stresses in a Future Climate

Any assessment of the impacts of increasing O_3_ exposure in ERs needs to consider effects of O_3_ alongside those of N deposition and climate change (Simpson, Arneth, Mills, Solberg, & Uddling, 2014), and alongside other environmental changes. Bobbink et al. (2010) assessed rates of N deposition in 2000, and under three scenarios by 2030, in the G200 ERs, comparing them with a generic effects threshold of 15 kg N ha^−1^ year^−1^. This analysis highlighted ERs in South‐East Asia as being under greatest threat. In particular, all the seven regions with the greatest increase in O_3_ exposure listed in Table 3a were identified as having high future N deposition. Based on implementation of current legislation to 2030, highest future N deposition is projected for Chota‐Nagpur dry forests and Terai‐Duar savannas and grassland. Bleeker, Hicks, Dentener, Galloway, and Erisman (2011) predicted that by 2030, 62 biodiversity hot spots and G200 ERs are projected to receive >30 kg N ha^−1^ year^−1^, with forest and grassland ecosystems in Asia most exposed.

Ozone exposure is expected to interact with N addition and/or warming, as reviewed by Mills et al. (2016). Effects of climate change on stomatal O_3_ flux and canopy uptake of O_3_ can be either direct—for example, temperature, CO_2_, and humidity effects on stomatal conductance—or indirect via an influence on soil water potential and plant development (Harmens, Mills, Emberson, & Ashmore, 2007; Mills et al., 2016). In addition, O_3_ itself can, for example, modify the responses of plants to naturally occurring environmental stresses such as drought (Hayes, Wagg, et al., 2012; Hayes, Williamson, et al., 2012; Mills, Hayes, Wilkinson, & Davies, 2009; Wilkinson & Davies, 2009, 2010) via effects on the hormonal control of stomatal functioning (Dumont et al., 2013) and plant development (canopy and roots), which can feedback to global warming (Sitch et al., 2007). Under O_3_ exposure, many species have smaller roots (Grantz et al., 2006), thereby enhancing drought sensitivity. Depending on species, O_3_ might induce stomatal closure, increased stomatal opening or sluggishness (Hoshika, Omasa, & Paoletti, 2013; Hoshika, Katata, et al., 2015), or have no effect (Mills et al., 2016). Differences in the specific response to O_3_ of stomatal control may thus affect species composition indirectly through variable soil moisture changes (Jäggi & Fuhrer, 2007). With progressive global climate change, drought episodes are projected to become more frequent in many world regions, and subtle interactions of O_3_ with water flux regulation may thereby influence community dynamics and species dominance.

Sun et al. (2012) suggested that loss of stomatal sensitivity in a Southern Appalachian forest in the USA will not only increase drought severity in the region, thus affecting ecosystem hydrology and productivity, but it will also have negative implications for flow‐dependent aquatic biota. When occurring over sufficiently large areas, high O_3_ effects on stomata could shift catchment water balances through altered canopy water fluxes (Lombardozzi, Levis, Bonan, Hess, & Sparks, 2015; McLaughlin, Wullschleger, Sun, & Nosal, 2007; Sun et al., 2012), with possible implications for the surface energy balance (Super, Vilà‐Guerau De Arellano, & Krol, 2015).

Some of the Asian regions such as the Tibetan plateau have also been identified as a hot spot of climate change impacts, both in terms of recent observed change (Shen et al., 2015; Turco, Palazzi, von Hardenberg, & Provenzale, 2015) and model projections (Diffenbaugh & Giorgi, 2012). Combining projections for both mean changes in temperature and precipitation with changes in the interannual variability of these parameters, simulations by Li et al. (2013) revealed that by the end of the 21st century, 96% of G200 ERs will face moderate to pronounced climatic changes relative to the change in the past five decades, with ERs at high northern latitudes being most exposed to change, followed by those in the Mediterranean Basin, Amazon Basin, East Africa, and South Asia. Hence, some of the priority ERs, which are highlighted in our analysis as being of greatest threat from increased O_3_ exposure, are also at high risk from N deposition and climate change, emphasizing the need to assess the effects of O_3_ together with other key components of environmental change.

Increasing CO_2_ in controlled environments or open‐top chambers often ameliorates effects of O_3_ on leaf physiology, growth, and C allocation; however, evidence from field‐based experiments does not support that they have fully compensatory effects when co‐occurring (Mills et al., 2016). Combined responses to elevated temperature and O_3_ have rarely been studied even though some critical growth stages such as seed initiation are sensitive to both. Kasurinen et al. (2012) showed that O_3_ modifies the response of temperate silver birch to warming, but the magnitude of response varies among genotypes.

Although the review by Mills et al. (2016) provides information on combined effects on plant processes, little information is available on combined effects of O_3_ and N on biodiversity. To our knowledge, only one experiment has studied the long‐term effects of combinations of O_3_ and N on biodiversity and plant processes in perennial grassland under field conditions (Bassin et al., 2013; Volk et al., 2014). Under climatically challenging conditions, added N to low background N deposition caused large changes in the community composition, with sedges becoming particularly dominant, while added O_3_ had no effect on functional group composition and few effects on productivity (see above). In Mediterranean annual grassland, N addition could partially counterbalance O_3_ effects on aboveground biomass in a mixture with six annual pasture species, but only when the levels of O_3_ were moderate, but at the same time, O_3_ reduced the fertilization effect of higher N availability (Calvete‐Sogo et al., 2014). Under the same conditions, a significant interaction between O_3_ and N input was found, where O_3_ caused a decline in the fraction of legumes while forbs and grasses proved to be tolerant, in contrast to the response to N (Calvete‐Sogo et al., 2016). Mills et al. (2016) concluded that it is not always straightforward to predict the direction of O_3_ effect once one or more interacting factors are included and that there is evidence of tipping points occurring where there is a shift from one factor being dominant to another. This shift can be dynamic and change during the growing season. Both responses to gradual changes in pollutants and climate and those under extreme weather events require further study.

## 9 Extrapolation

The majority of experimental studies reviewed above were carried out in temperate or Mediterranean climates of the NH. Hence, any global assessment of current and future O_3_ risks relies on extrapolation from limited experimental data. We thus distinguish predictions of impacts in those biomes, in which the impacts of O_3_ have hardly been investigated at all (e.g., tropical and subtropical forests or deserts and xeric shrublands), from those ERs within biomes that have been well studied in Europe and North America (e.g., broadleaf forests, Mediterranean grasslands, and montane grasslands). There is some evidence of comparable responses within genus between the two regions; for example, Hosika, Watanabe, et al. (2015) reported similar effects of O_3_ on stomatal behavior of European and Japanese beech species, while Hu et al. (2015) found that poplar clones grown in China were comparable in sensitivity to European beech and birch. However, few such studies, providing a direct comparison of sensitivity, have yet been reported.

Our assessment of the O_3_ exposure of ERs used a concentration‐based exposure index. There is increasing evidence that O_3_ effects are better related to the flux through the stomata into the leaves (Anav et al., 2016; Mills, Hayes, et al., 2011; Mills, Pleijel, et al., 2011), and hence, our assessment of the global effects should consider stomatal conductance as a key factor influencing the flux into and hence effect on different species. To date, no global assessment based on O_3_ flux is available, although total dry deposition of O_3_ has been modeled; Hardacre, Wild, and Emberson (2015) predicted that, at the same atmospheric concentration, O_3_ dry deposition was greater to tropical forests than to deciduous or coniferous forests, with deposition to tundra and deserts being the lowest. On this basis, tropical and subtropical forests may be relatively sensitive to O_3_, although not all of the modeled dry deposition would be stomatal uptake. But measurements of both AOT40 and stomatal O_3_ uptake in stands of *Schima superba* in subtropical China revealed seasonal exposures above current critical thresholds (Niu et al., 2016), thus confirming a potential ecological O_3_ risk in this region. Because of the importance of climate for leaf gas exchange, our extrapolation relies on the assumption that under comparable climatic conditions, O_3_ flux and related O_3_ risk would be similar for the same genus in different ERs. Intuitively, this could be the case for temperate broadleaf and mixed forest that have been well researched in Europe and also occur in the Eastern and Western Himalayas, for which relatively large increases in O_3_ exposure by 2050 is projected (Table 3). Linking stomatal conductance to plant functional types, as done by Lin et al. (2015), could help to extrapolate O_3_ uptake as a proxy for O_3_ sensitivity across biomes.

Resilience to O_3_ could be expected in the Eastern Himalayan alpine meadows and on the meadows and steppe on the Tibetan Plateau, similar to observations in European studies (see above); hence, increased O_3_ exposure projected for these higher altitude ERs of Asia (Table 3) would ecologically be less relevant. The high O_3_ sensitivity of annual grasslands is likely confined to Mediterranean regions of Europe and parts of California, where they have been the subjects of extensive research.

## 10 Conclusion: Implications of Different Climate and Air Pollution Policies

In spite of the limited direct evidence for O_3_ effects on terrestrial biodiversity, and of sufficient experimental and observational data from the full global range of ERs with high conservation value, the information presented in this study leads us to conclude that O_3_ levels are sufficiently high today, or will become so in the future, to exert a large‐scale influence on community composition at different trophic levels, and to alter nutrient and C cycling with possible feedbacks to the climate. Knowledge of the impacts of high O_3_ exposures in temperate forests is relatively strong, based primarily on work in North America and Europe, and there is also a good understanding of O_3_ impacts on ecosystem structure and dynamics in temperate grasslands and Mediterranean systems, primarily from work in Europe. This provides a basis for expecting clear ecological benefits within these biomes from reduced O_3_ exposures where these are simulated under the climate stabilization policy represented by RCP4.5. However, such changes are likely to be slow and may take decades to become detectable.

Our CESM simulations also reveal important differences in O_3_ trajectories, both between biomes and between individual ERs. Our analysis highlights a contrast between ERs in North America, where decreased exposure is predicted under both RCP4.5 and RCP8.5, and those in South and central Asia, where further increases in exposures are expected under both RCP4.5 and RCP8.5. Thus, even the emission projections associated with air quality and climate stabilization policies represented in RCP4.5 do not lead to a reduction in ecological O_3_ risks in many ERs, which are critical contributors to global biodiversity. Furthermore, under the RCP8.5 scenario that does not stabilize climate, O_3_ concentrations are likely to be significantly higher in the majority of ERs, especially in Asia, where ecological consequences are unclear (Koike et al., 2013).

Unfortunately, the ERs where the greatest increases in O_3_ levels are projected have not been investigated for possible O_3_ effects, and thus, the implications of these simulated trajectories are difficult to predict. Nevertheless, based on the evidence from Europe and North America, in moist ERs such as forests in the Western and Eastern Himalayas, on the Eastern Deccan Plateau in India, or the Terai‐Duar tropical and subtropical savanna and grasslands at the base of the Himalayas, future increases in O_3_ stress could alter C and N cycling and change inter‐ and intraspecific diversity. In contrast, from the evidence of resilience in montane grasslands in Europe, we predict that the Himalayan grasslands are at lower risk from increasing O_3_. Given the potential for negative effects in these species‐rich systems, more research is urgently needed.

Whether or not observed species shifts in temperate forests of North America can be extrapolated further, for example to forests in tropical and subtropical regions, remains very uncertain. However, because the initial biochemical and physiological reactions caused by excess O_3_ uptake are likely to be universal, the pattern of downstream effects on plants, host–pest interactions, and soil microbiota may be common to all biomes. This suggests that impacts on diversity at different trophic levels, with a range of potentially negative ecological consequences, are likely in ERs with increasing O_3_ exposures, even if the precise nature and the extent of these impacts cannot be predicted, and interactions with other global change factors such as N input and climate change are important. Finally, the evidence of altered trace gas fluxes under different O_3_ trajectories projections deserves more attention, as the consequent changes in climate forcing have implications for future assessments of cobenefits between climate and air pollution mitigation strategies.

## Conflict of Interest

None declared.
