# Supplementary material for: Current and future ozone risks to global terrestrial biodiversity and ecosystem processes
Source: Ecol Evol. 2016 Nov 21;6(24):8785–99. doi: 10.1002/ece3.2568 (PMC5192800; doi:10.1002/ece3.2568)
Supplement: Supplementary file 1 [file ECE3-6-8785-s003.pdf]

Appendix S1

Current and future ozone risks to global terrestrial biodiversity and ecosystem processes

Fuhrer, J. et al.

Community Earth System Model (CESM)

In this study, we used the global present-day (2000) and future 2050 hourly surface O_3 concentrations presented elsewhere (Val Martin *et al*., 2015) and previously used in the work of Tai *et al*. (2014). Specifically, we used the simulations from Val Martin *et al*. (2015) that include changes in climate forcing and emissions, but omit the effects of land cover and land use changes. These simulations were performed using the Community Earth System Model (CESM) v1.1.1 at a horizontal resolution of 1.9x2.5 degree, and vertical resolution of 26 layers from the surface to about 4 hPa, with a time step of 30 min. Our simulations use an optimized dry deposition scheme (Val Martin *et al*., 2014), which improves the simulation of O_3 dry deposition velocity and significantly reduces the well-known, long lasting summertime surface O_3 bias over eastern US and Europe documented by Lamarque *et al.* (2012). The CESM1.1.1 simulations have been extensively evaluated by comparison with satellite, sonde, aircraft and ground observations of O_3 on a global scale (Val Martin *et al*., 2014, Tilmes *et al*., 2016). Our CESM simulations represent well the spatial distribution, seasonality and magnitude of surface O_3, reproducing within 15% the observations throughout the lower troposphere. On a regional scale, the model overestimates summertime surface O_3 over the northern hemisphere mid and high latitudes, and slightly underestimates it over the southern hemisphere mid-latitudes (Tilmes *et al*., 2016). The simulations used coupled atmosphere and land components, and prescribed sea-surface and sea-ice distributors, corresponding to previous fully coupled simulations (Meehl *et al*., 2012). The model configuration also considered time varying, zonally averaged greenhouse gas distributions for CO_2, CH_4, N_2O and halogens, and future changes in stratospheric O_3 levels.

Anthropogenic emissions of greenhouse gases and O_3 precursors followed the RCP4.5 and RCP8.5 scenarios represented in IPCC AR5 (van Vuuren *et al*., 2011). These two RCP scenarios project decreases in most O_3 precursor emissions globally due to measures to abate air pollution, with the exception of CH_4, which increases (Table A1). Reductions in global emissions of 15/18% for CO, 13/24% for NO_x and 1/9% for volatile organic compounds (VOCs) are projected in 2050 compared to 2000 in RCP4.5 and RCP8.5, respectively. In the case of CH_4, global mean concentrations are projected to increase from a marginal 4% in RCP4.5 to a 56% in RCP8.5 by 2050.

Table S1. Summary of main global O_3 precursor emissions and CH_4 concentrations for 2000, and for 2050 under RCP4.5 and RCP8.5 scenarios.

Year	Scenario	CO (Tg CO yr^{-1})	NO_x (Tg NO_2 yr^{-1})	VOCs (Tg C yr^{-1})	CH_4 (ppb)
2000	Baseline	1069	127	211	1760
2050	RCP4.5	873	96	191	1833
	RCP8.5	906	110	208	2740

O_3 exposure indices

From the CESM simulations of hourly surface O_3 concentrations, we derived the 12-hour (daylight hours represented by 06:00-18:00) mean O_3 concentration (M12) averaged over each three-month period. For the purpose of comparison, we also calculated AOT40 over the same 3-month period; this is the index used by the UNECE Convention on Long-Range Transboundary Air Pollution (CLRTAP) to define Critical Levels to protect the most sensitive species, or of (semi-)natural communities dominated by annuals (CLRTAP, 2015).

$$\mathrm{M12\ (ppb)} = \left(\sum_{i=1}^{n}[Co_3]_i\right)/n$$

$$\mathrm{AOT40\ (ppm.h)} = \sum_{i=1}^{n}([Co_3]i - 0.04)\ \mathrm{for}\ Co_3\ \geq 0.04\ \mathrm{ppm}$$

where

$[Co_3]$ is the hourly mean O_3 concentration during daylight hours (represented by 06:00-18:00), and n is the number of hours in each three-month-period.

For the purpose of comparison, Figure A1 shows the relationship between maximum M12 and the maximum AOT40 value based on simulations for all G200 terrestrial ecoregions in 2000.

The relationship was fitted with the quadratic polynomial function:

AOT40 = 9.33-0.839M12+0.0184(M12)^2 (R^2=0.963).

The dashed line indicates the AOT40 value of 3ppm.h, set by the CLRTAP as the concentration-based Critical Level to protect the most sensitive species of semi-natural communities dominated by annual species (CLRTAP, 2015). However, we did not use the simulated AOT40 directly in our global assessment, because of the lower performance of such modelled cumulative indices, compared to those based on mean concentrations such as M12.

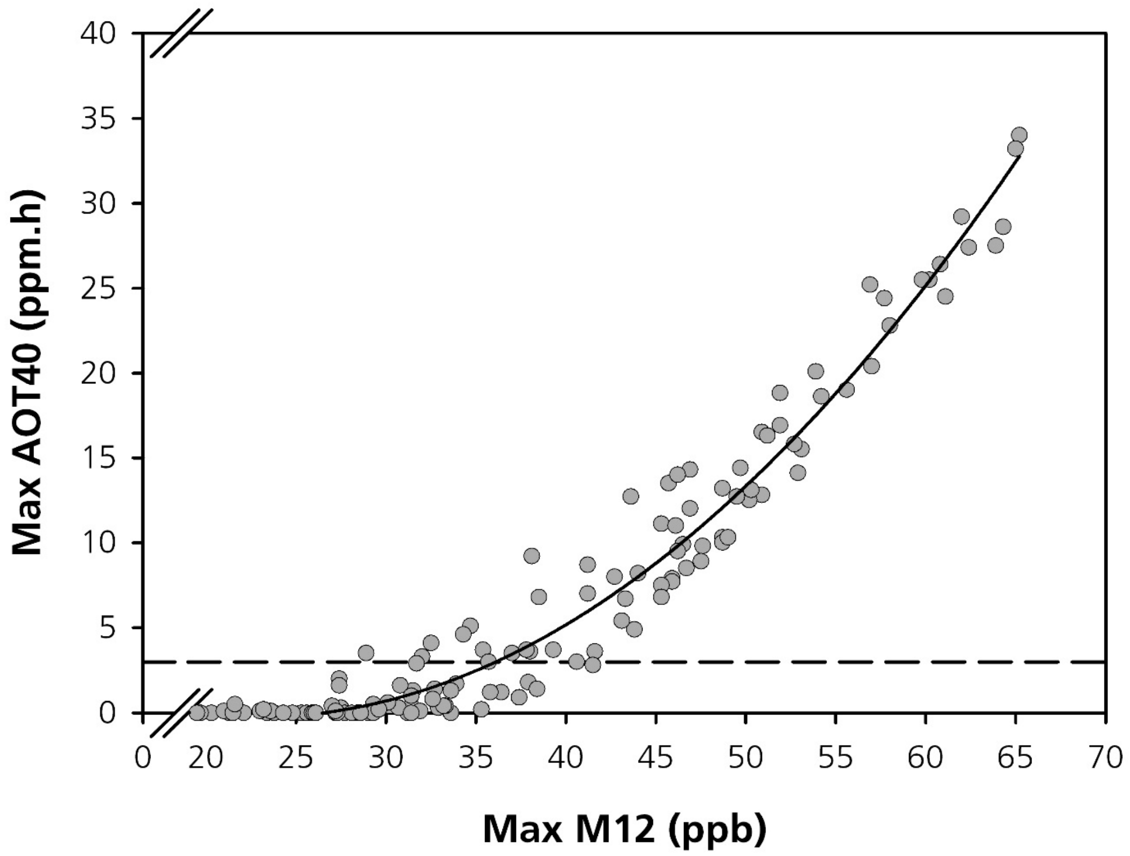

Figure S1. Relationship between simulated maximum M12 and maximum AOT40 across all G200 ecoregions.

Records of visible injury

While identification of visible O_3 injury can be difficult, due to the potential for similar symptoms caused by drought, insects, natural senescence, or pathogens (Bussotti *et al.*, 2003), steps can be taken to ensure that symptoms are assessed as accurately as possible. Although injury symptoms can vary between plant species, there are several diagnostic features that tend to be commonly found in O_3-damaged plants: 1) Spotting on the leaves occurs between the leaf veins; 2) Damage appears on the upper surface of the leaves, spreading to the underside in severe cases; 3) Older leaves (towards the base of the stem and branches) tend to be more affected than younger leaves as damage is determined by the accumulated uptake of O_3 over time (ICP Vegetation, 2016). These symptoms have been confirmed for a number of species used in biomonitoring experiments under controlled conditions (e.g. Burkey *et al.*, 2005, Orendovici *et al.*, 2003). Handbooks and manuals containing guidance on identifying symptoms and photographs of O_3 injury in a variety of species are available (Innes *et al.*, 2001,

Kohut 2005, Schaub *et al.,* 2010). Microscopy can also be used to confirm O_3 injury (e.g. Günthardt-Goerg *et al.,* 2000), if necessary.

Records of visible O_3 injury on forbs, shrubs and trees occurring under ambient conditions between 2007 and 2015 were gathered from a number of sources: a) by searching the peer-reviewed literature using Web of Science in August 2015 with the terms "visible O_3 injury", "foliar injury and O_3", "ethylenediurea and O_3" (EDU experiments tend to record visible injury symptoms on vegetation in ambient air, comparing between control plants and those treated with EDU); b) by using data from ICP Vegetation biomonitoring experiments carried out with white clover (*Trifolium repens*) and bean (*Phaseoulus vulgaris*) (Mills *et al*., 2011); and c) using data from the new ICP Vegetation smart-phone O_3 injury app (Harmens *et al*., 2015). Records represent the presence of injury at a site in a particular month or year (i.e. the number of individual injured plants has not been counted). If repeated observations of injury were made on the same set of plants during a growing season, only the first incidence of injury was counted. Studies published after 2006 but with no information on the year that the experiment was carried out, or that symptoms observed, were not included.

It is important to document how the visible injury data has been verified for each source. Data on injury was taken from 19 sources in the peer-reviewed literature. Of these, 8 used microscopy (where necessary) to confirm injury symptoms, 7 describe the use of manuals (for example, the ICP Forest manual; Schaub *et al*., 2010) and photographs of O_3 symptoms, confirmed experimentally, while 4 do not directly describe injury validation, however were carried out at institutions with years of experience of investigating O_3 damage to vegetation. The data from North America (244 records) were gathered as part of an O_3 biomonitoring program by the US Dept. Agriculture Forest Service, with injured leaves from every location sent to an expert O_3 diagnostician for validation. ICP Vegetation biomonitoring data was gathered by experts, following a protocol, which includes example photographs of injured leaves. For the bean experiments, as the first true leaves are susceptible to environment and biotic stress, O_3 injury symptoms were only recorded on trifoliate leaves. Photographs of the suspected injury were also submitted to the ICP Vegetation Programme Coordination Centre (CEH Bangor, UK; http://icpvegetation.ceh.ac.uk) for verification. When users submit records of O_3 injury using the new smart phone App, they are guided through a series of questions about the symptoms, designed to aid identification of true injury. The App also contains information sheets and photographs outlining the symptoms of other sources of leaf injury, for example, insect damage or disease. When records are submitted, they are verified by experts at the ICP Vegetation Programme Coordination Centre.

Ecoregion O_3 exposure

Table S2. Maximum values of M12 (ppb) for each ecoregion for 2000 and for 2050 under RCP4.5 and RCP8.5.

**Ecoregion (ER)**	**2000**	**2050**	**2050**
	**Baseline**	**RCP45**	**RCP85**
Appalachian and Mixed Mesophytic Forests	65.2	48.5	54.9
California Chaparral and Woodlands	65.0	52.5	56.9
Western Himalayan Temperate Forests	64.3	73.3	78.3
Tibetan Plateau Steppe	63.9	69.0	76.1
Terai-Duar Savannas and Grasslands	62.4	73.4	75.8
Sierra Nevada Coniferous Forests	62.0	49.6	56.0
Eastern Himalayan Alpine Meadows	61.1	68.7	73.3
Mediterranean Forests	60.8	57.9	64.1
Caucasus-Anatolian-Hyrcanian Temperate Forests	60.2	57.4	64.5
Middle Asian Montane Steppe and Woodlands	59.8	57.7	63.9
Sonoran-Baja Deserts	58.0	45.9	51.3
European-Mediterranean Montane Mixed Forests	57.7	52.9	59.2
Eastern Himalayan Broadleaf and Conifer Forests	57.0	65.2	68.8
Chhota-Nagpur Dry Forests	56.9	68.5	68.5
Hengduan Shan Coniferous Forests	55.6	59.3	65.8
Central Asian Deserts	54.2	50.9	57.3
Southeastern Coniferous and Broadleaf Forests	53.9	41.4	46.0
Altai-Sayan Montane Forests	53.1	49.7	56.0
Nansei Shoto Archipelago Forests	52.9	54.0	60.3
Daurian Steppe	52.7	49.2	55.2
Eastern Deccan Plateau Moist Forests	51.9	61.7	61.7
Southwest China Temperate Forests	51.9	47.1	56.6
Northern Prairie	51.2	46.0	54.2
Taiwan Montane Forests	50.9	52.2	58.4
Naga-Manupuri–Chin Hills Moist Forests	50.9	52.9	56.8
Everglades Flooded Grassland	50.3	42.0	46.2
Russian Far East Temperate Forests	50.2	48.2	53.3
Rann of Kutch Flooded Grasslands	49.7	53.1	58.1
Klamath-Siskiyou Coniferous Forests	49.5	47.3	53.8
Taimyr and Siberian Coastal Tundra	49.0	47.1	51.1
Eastern Siberian Taiga	48.7	46.3	51.4
Kamchatka Taiga and Grasslands	48.7	45.0	50.0

Chihuahuan-Tehuacan Deserts	48.7	41.5	49.0
Pacific Temperate Rainforests	47.6	47.1	52.4
Chukote Coastal Tundra	47.5	46.0	49.4
Northern Indochina Subtropical Moist Forests	46.9	43.8	52.4
Sudanian Savannas	46.9	50.4	52.0
Canadian Low Arctic Tundra	46.7	45.3	49.2
Mexican dry Forests	46.5	43.5	48.7
Sierra Madre Oriental and Occidental Pine/Oak Forest	46.2	42.1	48.7
Sudd-Sahelian Flooded Grasslands and Savannas	46.2	49.9	51.3
Sundarbans Mangroves	46.1	50.0	52.6
Muskwa/Slave Lake Boreal Forests	45.9	45.5	49.9
Canadian Boreal Forests	45.9	44.5	48.8
Cameroon Highland Forests	45.7	50.1	52.6
Pantanal Flooded Savannas	45.3	42.1	48.7
Alaskan North Slope Coastal Tundra	45.3	43.9	47.4
Fenno-Scandia Alpine Tundra and Taiga	45.3	45.6	50.4
Southeast China-Hainan Moist Forests	44.0	40.1	45.7
Annamite Range Moist Forests	43.8	42.5	49.8
Chiquitano Dry Forests	43.6	36.3	43.6
Arabian Highland Woodlands and Shrublands	43.3	48.8	52.7
Ural Mountains Taiga	43.1	41.7	46.9
Mesoamerican Pine-Oak Forests	42.7	40.1	43.4
Indochina Dry Forests	41.6	40.3	47.2
Socotra Island Desert	41.5	48.2	50.4
Guinean Moist Forest	41.2	47.5	49.3
Kayah-Karen/Tenasserim Moist Forests	41.2	40.1	46.0
Cardamom Mountains Moist Forests	40.6	39.9	46.2
Southwestern Ghats Moist Forests	39.3	46.4	47.5
Zambezian Flooded Savannas	38.5	37.0	41.2
Greater Antillean Pine Forests	38.4	39.9	44.7
Northeastern Congo Basin Moist Forests	38.1	44.3	42.5
Peninsular Malaysian Lowland and Montane Forest	38.0	40.9	35.5
Hawaii Moist Forests	37.9	32.3	36.8
Ethiopian Highlands	37.8	43.7	45.0
Greater Antillean Moist Forests	37.4	39.5	44.1
Western Java Montane Forests	37.0	38.5	30.0
Hawaii Dry Forests	36.4	30.6	35.3

Central Andean Dry Puna	35.8	34.7	39.4
Cerrado Woodlands and Savannas	35.7	34.5	39.0
Albertine Rift Montane Forests	35.4	39.9	41.7
Sri Lankan Moist Forests	35.3	34.5	40.0
Central and Eastern Miombo Woodlands	34.7	33.7	37.1
Gulf of Guinea Mangroves	34.3	43.3	43.8
Drakensberg Montane Shrublands and Woodlands	33.9	33.6	39.2
Coastal Venezuela Montane Forests	33.6	34.5	38.2
Namib-Karoo-Kaokoveld Deserts	33.6	33.8	38.7
Horn of Africa Acacia Savannas	33.3	38.0	39.6
Great Sandy-Tanami Deserts	33.2	32.4	35.7
Palawan Moist Forests	32.9	32.4	35.5
Sumatran Islands Lowland and Montane Forest	32.7	33.4	29.5
Atacama-Sechura Deserts	32.6	31.7	35.7
Southwestern Amazonian Moist Forests	32.5	29.1	35.8
Central Congo Basin Moist Forests	32.0	34.6	36.5
Chilean Matorral	31.9	32.6	37.0
Greater Sundas Mangroves	31.7	28.0	28.3
Philippine Moist Forests	31.5	32.2	35.9
Choco–Darien Moist Forests	31.4	32.6	34.8
Northern Australia and Trans-Fly Savannas	31.4	30.5	32.5
Carnavon Xeric Scrub	31.4	30.1	33.9
Talamancan-Isthmian Pacific Forests	31.2	32.6	35.2
Central Andean Yungas	30.8	30.6	36.2
Llanos Savannas	30.7	32.5	35.7
Atlantic Forests	30.2	30.7	34.2
Southern Rift Montane Woodlands	30.1	29.8	32.6
Panama Bight Mangroves	29.6	31.1	32.5
East African Moorlands	29.3	36.8	35.5
Fynbos	29.3	31.6	35.4
Eastern Australia Temperate Forests	29.1	28.4	31.5
New Caledonia Moist Forests	29.0	31.3	34.3
New Caledonia Dry Forests	29.0	31.3	34.3
Western Congo Basin Moist Forests	28.9	32.6	33.3
Southern Australia Mallee and Woodlands	28.6	28.4	31.6
Atlantic Dry Forests	28.5	28.9	32.1
Queensland Tropical Forests	28.4	29.2	32.6
New Zealand Temperate Forests	28.1	30.5	33.7

Seychelle and Mascarene Moist Forests	27.7	31.0	34.3
Tasmanian Temperate Rain Forests	27.7	30.5	34.1
Northern Andean Montane Forests	27.5	28.8	30.9
Congolian Coastal Forests	27.4	29.2	32.0
Borneo Lowland and Montane Forests	27.4	22.8	24.6
Madagascar Mangroves	27.3	30.4	32.9
Nusa Tenggara Dry Forests	27.2	27.4	30.0
Kinabalu Montane Shrublands	27.2	25.7	25.1
Madagascar Spiny Thicket	27.2	30.0	33.2
Guianan–Amazon Mangroves	27.2	29.1	31.4
East African Acacia Savannas	27.0	30.5	32.8
East African Mangroves	26.1	29.0	31.2
Southwestern Australia Forests and Scrub	26.0	25.7	28.7
Madagascar Dry Forests	25.9	29.0	31.5
Madagascar Forests and Shrublands	25.6	28.6	31.5
Eastern Arc Montane Forests	25.3	28.0	30.1
Valdivian Temperate Rainforests/Juan Fernandez Island	24.8	27.4	30.5
Patagonian Steppe	24.3	27.0	29.8
Guayanan Moist Forests	23.8	26.4	28.4
Tumbesian-Andean Valleys Dry Forests	23.6	23.5	26.2
East African Coastal Forests	23.4	26.3	27.8
Northern Andean Paramo	23.2	22.9	26.0
Guayanan Highland Moist Forests	23.0	25.3	27.7
South Pacific Island Forests	22.1	23.7	25.4
Amazon River and Flooded Forests	21.6	21.2	24.5
Galapagos Islands Scrub	21.5	21.2	22.9
Sulawesi Moist Forests	21.3	20.0	20.5
Napo Moist Forests	21.0	20.4	24.0
Moluccas Moist Forests	20.3	17.1	18.5
Southern New Guinea Lowland Forests	19.7	14.3	17.4
New Guinea Mangroves	19.5	14.7	17.3
Rio Negro-Jurua _ Moist Forests	16.6	17.8	19.2
New Guinea Montane Forests	16.5	16.8	17.9
Solomons-Vanuatu-Bismarck Moist Forests	15.8	15.5	16.8

Extended simulation results

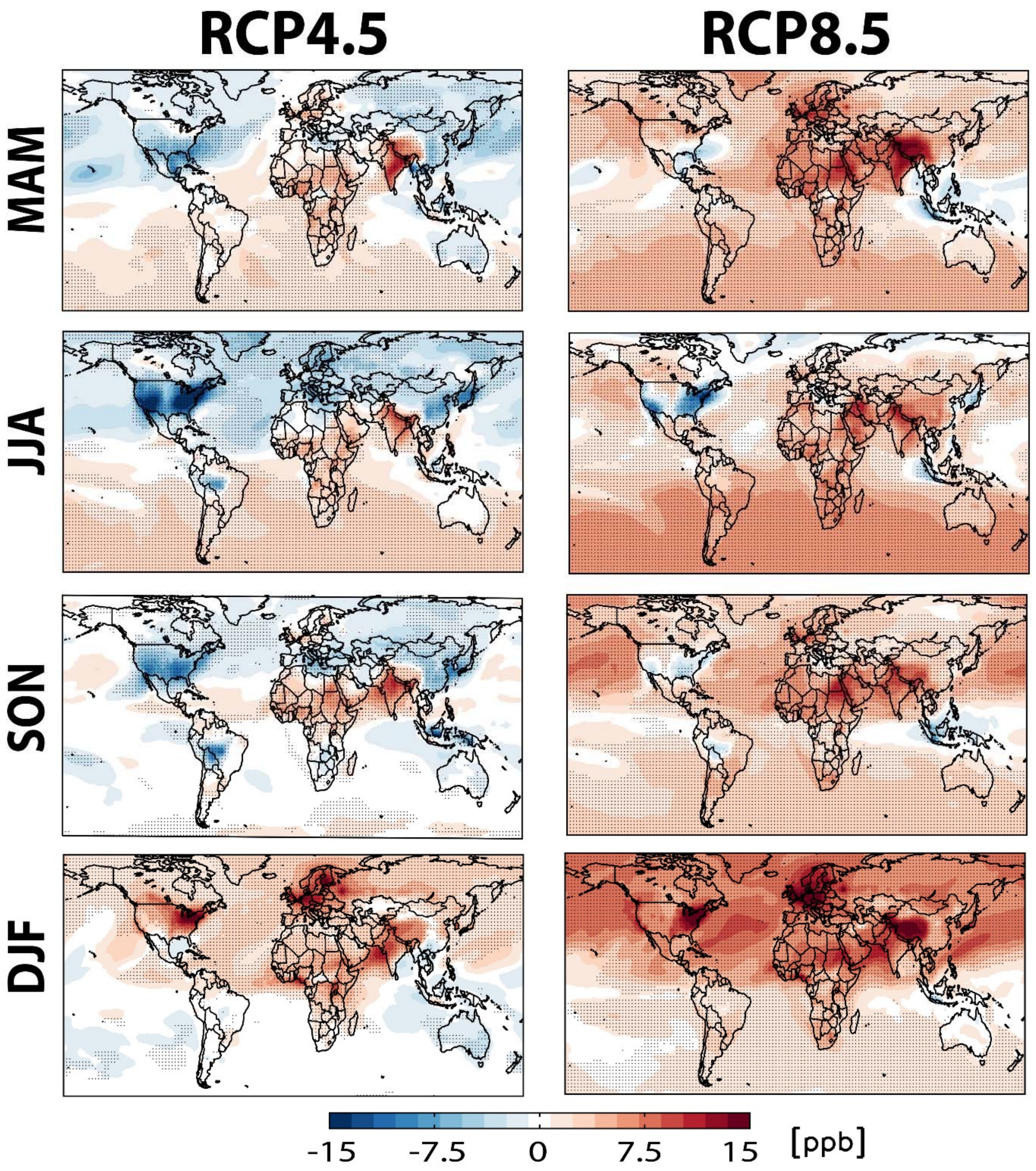

Figure S2. Projected changes of O3 concentration between 2000 and 2050 as a result of the combination of climate and emission changes for RCP4.5 and RCP8.5. Maps show interpolated contours from the 1.9x2.5 degree horizontal resolution output. Reading downwards, the Figure shows changes in seasonal M12 values for March-April-May (MAM), June-July-August (JJA), September-October-November (SON) and December-January-February (DJF). Regions with changes that are significant at the 95% confidence level are indicated with dots.

Changes in O_3 exposure due to either climate or precursor emission

Table S3. Changes in O_3 concentrations (ppb) 2050-2000 in G200 terrestrial ecoregions, grouped by biome. Values are based on the highest monthly M12 value (see main text). Tabulated values are for the mean change in M12 value within a biome and the range of values (minimum-maximum) in individual ecoregions within that biome. The changes in M12 are split between those due to changes in precursor emissions and those due to climate change.

	RCP4.5				RCP8.5			
**Major biome**	**M12 2050**	**Overall change 2050-2000**	**Change due to emissions**	**Change due to climate**	**M12 2050**	**Overall change 2050-2000**	**Change due to emissions**	**Change due to climate**
Tropical and subtropical moist broadleaf forests	33.7 (15.8-52.9)	1.1 (-5.6–9.8)	0.2 (-5.7–8.8)	0.8 (-4.4–4.6)	36.3 (16.8–61.7)	3.7 (-7.0–9.8)	2.5 (-6.5–9.3)	1.2 (-4.5–3.9)
Tropical and subtropical dry broadleaf forests	37.4 (23.6-56.9)	-0.4 (-7.3–11.5)	-0.7 (-7.6–9.3)	0.3 (-4.7–2.3)	41.1 (26.2–68.5)	3.7 (-1.1–11.5)	2.7 (-1.3–9.5)	0.9 (-4.5–2.4)
Temperate broadleaf and mixed forests	46.7 (27.7-65.2)	-0.2 (-16.7–9.0)	-2.3 (-21.7–6.1)	2.1 (-0.1–5.0)	51.4 (31.5–78.3)	4.7 (-10.3–14.0)	1.9 (-16.7–10.6)	2.9 (-0.2–6.4)
Temperate coniferous forests	51.6 (24.8-62.9)	-3.6 (-12.5–3.7)	-5.2 (-15.8–1.5)	1.6 (-1.3–3.3)	53.8 (30.5–65.8)	2.2 (-7.9–10.2)	-0.2 (-11.0–6.8)	2.4 (-0.4–4.6)
Boreal forests/taiga	46.5 (43.1-48.7)	-1.9 (-3.7–-0.4)	-1.4 (-1.6–-1.3)	-0.4 (-2.2–1.1)	49.4 (46.9–51.4)	2.9 (1.3–3.9)	3.2 (2.9–3.5)	-0.3 (-2.2–0.9)
Tropical and subtropical grasslands, savannas and shrublands	37.8 (27.0-62.4)	2.7 (-1.1–11.1)	1.3 (-1.9–7.9)	1.4 (-0.2–3.1)	43.1 (32.5–75.8)	5.3 (1.1–13.4)	4.1 (1.6–10.6)	1.2 (-0.4–2.8)
Temperate grasslands, savannas and shrublands	44.8 (24.3-52.7)	-1.5 (-8.3–3.7)	-2.1 (-7.2–2.6)	0.6 (-1.7–2.3)	48.1 (29.8–58.1)	3.3 (-4.0–8.4)	2.4 (-1.9–6.9)	1.0 (-2.1–3.4)
Montane grasslands and shrublands	40.2 (23.2-63.9)	2.1 (-2.1–7.6)	0.6 (-3.3–4.7)	1.5 (-1.4–3.6)	45.6 (25.1–76.1)	5.4 (-2.1–12.3)	3.9 (0.1–9.0)	1.5 (-2.2–3.7)
Tundra	46.8 (45.3-49.0)	-1.2 (-2.0–0.3)	-1.3 (-1.4–-1.1)	0.0 (-0.8–1.6)	49.5 (47.4–51.1)	2.7 (1.8–5.1)	3.4 (3.0–3.8)	-0.7 (-1.7–1.7)
Mediterranean forests, woodlands and shrubs	40.3 (26.0-65.0)	-2.2 (-12.5–2.3)	-3.6 (-13.3–0.4)	1.4 (0.7–2.5)	42.3 (28.7-64.1)	2.0 (-8.1–6.1)	-0.1 (-9.4–3.6)	2.2 (1.3–3.0)
Deserts and xeric shrublands	38.7 (21.5-58.0)	-1.0 (-12.1–6.7)	-1.8 (-11.6–5.2)	0.8 (-0.5–2.5)	41.9 (22.9–57.3)	3.2 (-6.7–9.3)	1.9 (-7.1–7.6)	1.3 (0.0–2.9)
Mangroves	29.3 (19.5-46.1)	1.5 (-4.8–9.0)	0.3 (-4.6–6.0)	1.2 (-2.1–4.6)	32.7 (17.3–52.6)	3.5 (-3.5–9.5)	2.4 (-2.8–7.1)	1.1 (-1.2–3.2)

References

Burkey KO, Miller JE, Fiscus EL (2005) Assessment of ambient ozone effects on vegetation using snap bean as a bioindicator species. *Journal of Environmental Quality*, **34**, 1081-1086.

CLRTAP (2015) Mapping Critical Levels for Vegetation, Chapter III of Manual on methodologies and criteria for modelling and mapping critical loads and levels and air pollution effects, risks and trends. UNECE Convention on Long-range Transboundary Air Pollution (accessed on 28-01-2016 on Web at www.icpmapping.org)

Harmens H, Mills G, Hayes F, Sharps K, Frontasyeva M. and the participants of the ICP Vegetation. ICP Vegetation Annual Report 2013/14. Programme Coordination Centre of the ICP Vegetation. Centre for Ecology & Hydrology, Bangor, UK. ISBN 978-1-906698-53-9

ICP Vegetation. Have you seen these Ozone Symptoms? Centre for Hydrology & Ecology, Bangor, UK (accessed 28-01-2016 on Web at

http://icpvegetation.ceh.ac.uk/publications/documents/CEHOzoneInjury_webmidres.pdf

Lamarque J-F, Emmons LK, Hess PG, Kinnison DE, Tilmes S, Vitt F, Heald CL, Holland EA, Lauritzen PH, Neu J, Orlando JJ, Rasch PJ, Tyndall GK (2012) CAMChem: description and evaluation of interactive atmospheric chemistry in the Community Earth System Model. *Geoscience Model Development*, **5**, 369–411.

Meehl GA, Hu A, Tebaldi C, Arblaster JM, Washington Warren M, Teng H, Sanderson BM, Ault T, Strand WG, White JB (2012) Relative outcomes of climate change mitigation related to temperature versus sea level and sea level rise. *Nature Climate Change*, **2**, 576–580.

Mills G, Hayes F, Simpson D, Emberson L, Norris D, Harmens H, Büker P (2011) Evidence of widespread effects of O3 on crops and (semi-)natural vegetation in Europe (1990-2006) in relation to AOT40- and flux-based risk maps. *Global Change Biology*, **17**, 592-613.

Orendovici T, Skelly JM, Ferdinand JA, Savage JE, Sanz M-J, Smith GC (2003) Response of native plants of northeastern United States and southern Spain to ozone exposures: determining exposure/response relationships. *Environmental Pollution*, **125**, 31-40.

Schaub M, Calatayud V, Ferretti M, Brunialti G, Lövblad G, Krause G, Sanz MJ (2010) Monitoring of Ozone Injury. Manual Part X. In: Manual on methods and criteria for harmonized sampling, assessment, monitoring and analysis of the effects of air pollution on forests. UNECE ICP Forests Programme Co-ordinating Centre, Hamburg. (accessed 28-01-2016 on Web at http://www.icpforests.org/Manual.htm).

Tai APK, Val Martin M, Heald CL (2014) Threat to future global food security from climate change and ozone air pollution. *Nature Climate Change*, **4**, 817-821.

Tilmes S, Lamarque J-F, Emmons LK, Kinnison DE, Marsh D, Garcia RR, Smith AK, Neely RR, Conley A, Vitt F, Val Martin M, Tanimoto H, Simpson I, Blake D R, Blake N (2016). Representation of the Community Earth System Model (CESM1) CAM4-chem within the Chemistry-Climate Model Initiative (CCMI). *Geoscientific Model Development*, **9**, 1853–1890.

U.S. Environmental Protection Agency (2014) Welfare Risk and Exposure Assessment for Ozone (accessed 28-01-2016 on Web at http://www3.epa.gov/ttn/naaqs/standards/ozone/data/20140829healthrea.pdf).

van Vuuren DP, Edmonds J, Kainuma M, Riahi K, Thomson A, Hibbard K, Hurtt GC, Kram T, Krey V, Lamarque JF, Masui T, Meinshausen M, Nakicenovic N, Smith SJ, Rose SK (2011) The representative concentration pathways: an overview. *Climatic Change*, **109**, 5-31.

Val Martin M, Heald CL, Arnold SR (2014) Coupling dry deposition to vegetation phenology in the Community Earth System Model: implications for surface O_3. *Geophysical Research Letters*, **41**, 2988-2996.

Val Martin M, Heald CL, Lamarque J-F, Tilmes S, Emmons LK, Schichtel BA (2015) How emissions, climate, and land use change will impact mid-century air quality over the United States: A focus on effects at National Parks. *Atmospheric Chemistry & Physics*, **15**, 2805-2823.
