# Supplementary figures and images for: Current and future ozone risks to global terrestrial biodiversity and ecosystem processes
Source: Ecol Evol. 2016 Nov 21;6(24):8785–99. doi: 10.1002/ece3.2568 (PMC5192800; doi:10.1002/ece3.2568)

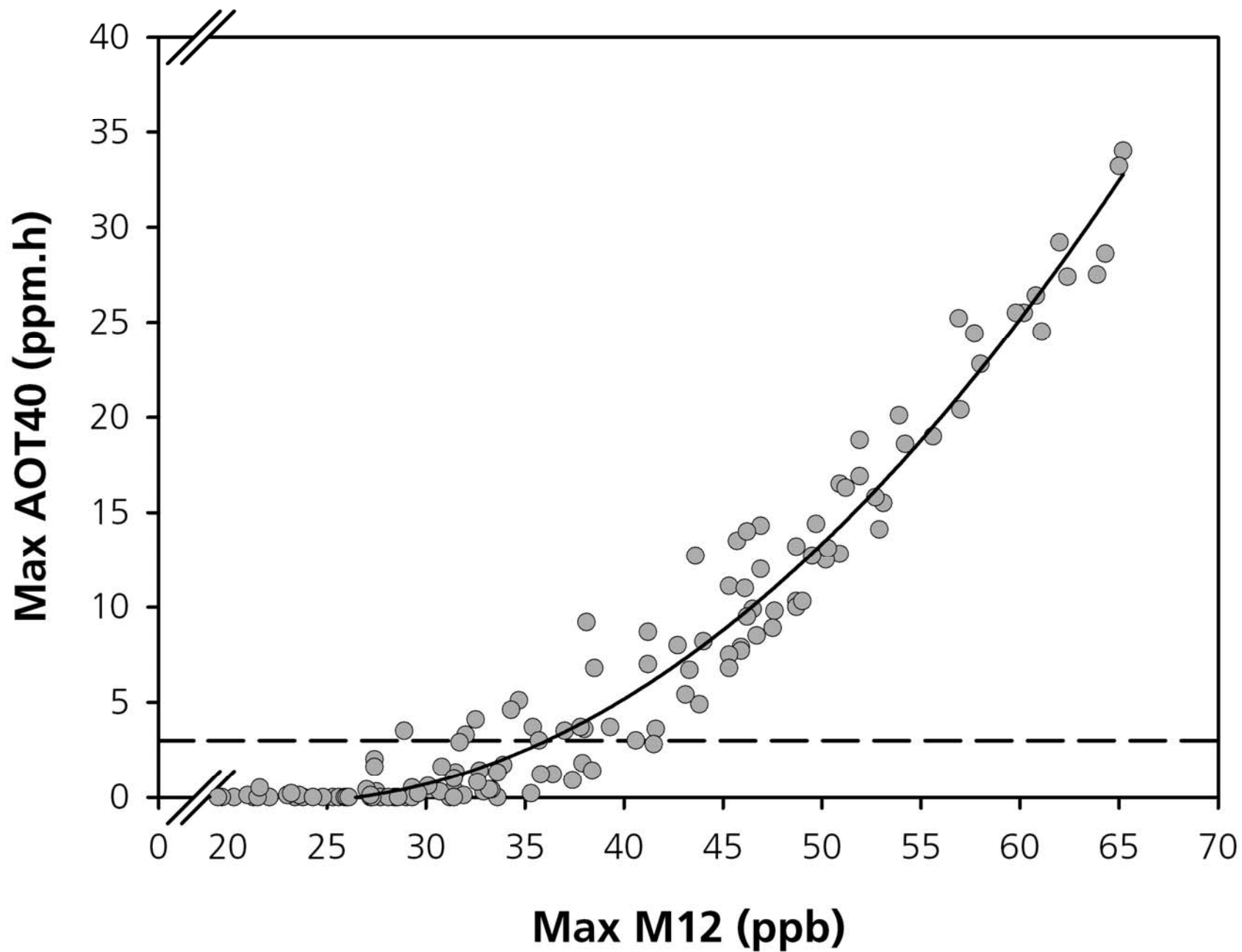

Supplement: Supplementary file 2 [file ECE3-6-8785-s001.pdf]

**RCP4.5**

**RCP8.5**

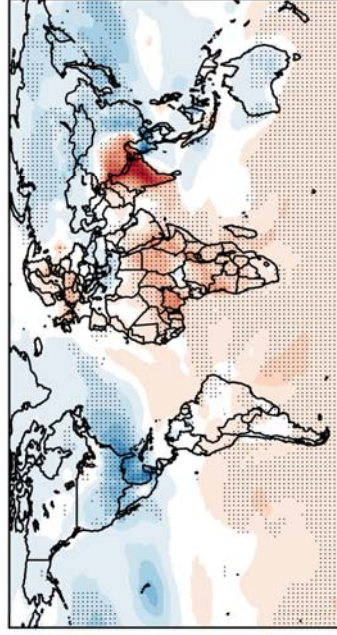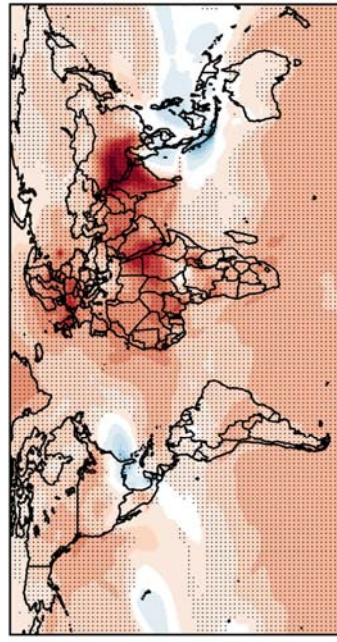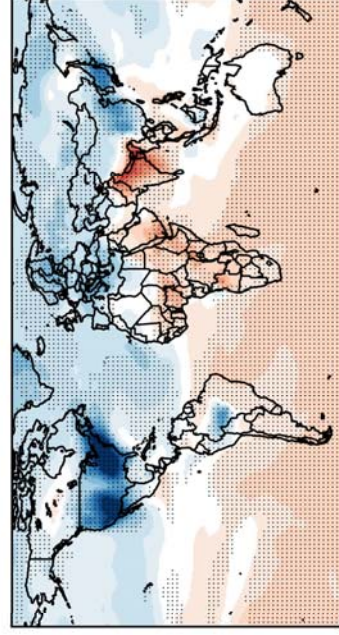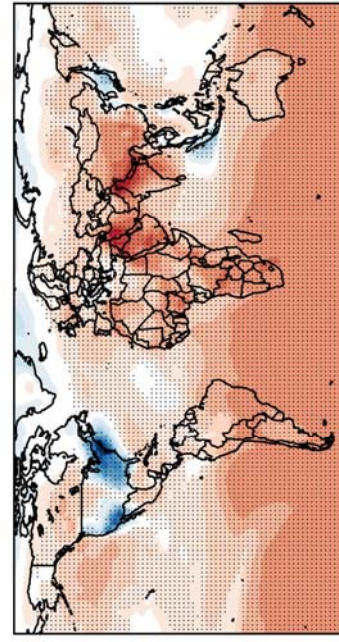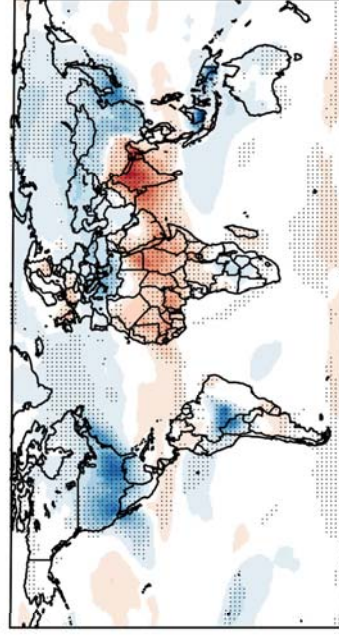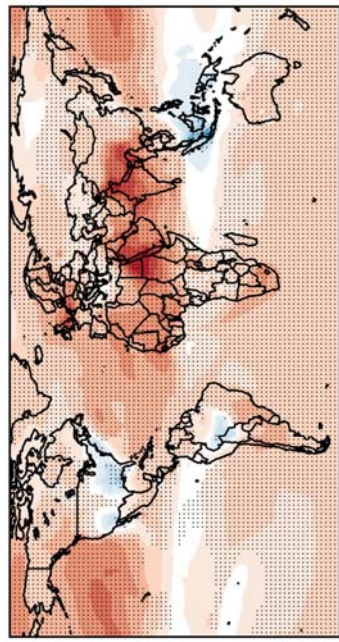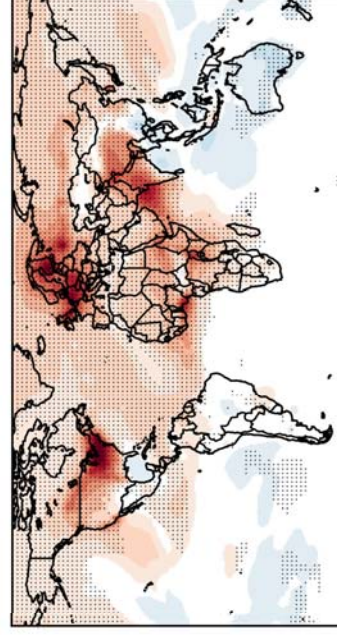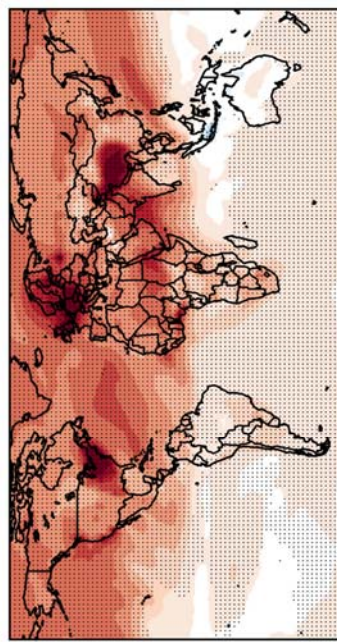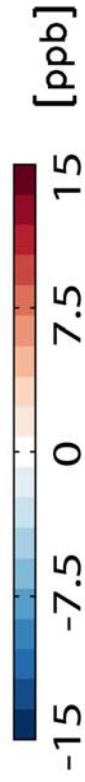

**MAM**

**JJA**

**SON**

**DJF**

Supplement: Supplementary file 3 [file ECE3-6-8785-s002.pdf]
